# Endoscope-assisted Transmastoid Infralabyrinthine approach to the petrous apex: A new corridor for multicompartmental jugular foramen tumors

**DOI:** 10.1016/j.bas.2026.105965

**Published:** 2026-02-09

**Authors:** Nobuyuki Watanabe, Sai Lok Chu, Norio Ichimasu, Arianna Fava, Jerold Justo, Jonathan Chainey, Tingting Jiang, Thibault Passeri, Luca Regli, Kentaro Watanabe, Sébastien Froelich

**Affiliations:** a Department of Neurosurgery, Lariboisière Hospital, Assistance Publique– Hopitaux de Paris, Paris, France; b Department of Neurosurgery, University Hospital of Zurich, Zurich, Switzerland; c Department of Neurosurgery, Jikei University School of Medicine, Nishishinbashi, Tokyo, Japan; d University of Paris, Paris, France

**Keywords:** Petrous apex, Endoscope-assisted, Transmastoid, Chondrosarcoma, Jugular foramen

## Abstract

**Introduction:**

Multicompartmental jugular foramen tumors may extend anteriorly and superiorly toward the petrous apex, posing significant challenges for traditional surgical corridors.

**Research question:**

The authors study the feasibility of endoscope-assisted transmastoid infralabyrinthine approach (e-TIA) to access the superior aspect of petrous apex.

**Materials and methods:**

Five cadaveric specimens were studied. A reference object was placed transnasally at the petrous apex to assess surgical exposure. To assess the working space of e-TIA, cross-sectional area was measured using three-dimensional reconstruction. Furthermore, we reviewed three patients with multicompartmental jugular foramen tumor in which this corridor was used.

**Results:**

The e-TIA, initiated with a 30° endoscope using the chopstick technique, accessed the superior petrous apex, Meckel's cave, and the internal carotid artery (ICA) from its cervical to lacerum segments. Radiological analysis demonstrated that the approach trajectory shifts at the cochlea level. Cross-sectional area analysis revealed that the e-TIA corridor provides sufficient space for manipulation of the endoscope, suction, and drill. Clinically, near total resection was achieved in all cases without new neurological complications.

**Discussion and conclusion:**

The e-TIA offers a systematic extension to the superior petrous apex and the ICA from its cervical to the lacerum segments. utilizing an angled endoscope to enhance visualization. When combined with an anterolateral approach, this dual-corridor strategy provides a unique “looking-up” line of sight to the petrous bone. Overall, the e-TIA represents a promising option in selected multicompartmental jugular foramen tumors with petrous apex extension.

## Highlights

• The e-TIA accesses the superior petrous apex, Meckel's cave, and the ICA from the cervical to lacerum segment.
• The corridor offers sufficient space for 30° endoscope, chopstick technique, simultaneous instrument use.
• Dual-corridor strategy with the anterolateral approach allows resection of tumors extending into the cervical portion.
• The e-TIA is a promising option for selected multicompartmental jugular foramen tumors with petrous apex extension.

## 1 Introduction

Lesions involving or extending into the petrous bone has posed a significant challenge for skull base surgeons due to its deep location and complex anatomy. The transpetrosal approach, which involves creating a surgical corridor by drilling portions of the petrous bone, is commonly employed to target intracranial lesions such as petroclival meningiomas or trigeminal schwannomas. However, this region also harbors bony tumors originating within the petrous bone itself. Multicompartmental jugular foramen tumors, such as large chondrosarcomas, may extend anteriorly and superiorly up to petrous apex. Critical sensory and neurovascular structures into the petrous bone, such as the semicircular canals and cochlea, represent an obstacle to access the most anterior and superior part of these rare tumors when using a classic transmastoid approach precluding a complete resection that may require an additional surgical stage. Some studies have examined modifications of the transmastoid approach to reach the petrous apex or petroclival region; however, the risk of facial nerve injury continues to pose a significant concern (Cinibulak et al., 2013, 2022, 2024a, 2024b; Miller et al., 2016; Llorente et al., 2014; Nonaka et al., 2013).

To enhance the utility of the transmastoid approach for accessing the most anterior and superior aspect of the petrous pyramid, we have incorporated the use of an angled endoscope through the transmastoid infralabyrinthine corridor. This concept can facilitate access to the superior aspect of the petrous apex and Meckel's cave through a transmastoid corridor, without compromising critical structures. We present an anatomical study using five cadaveric specimens and three surgical cases to illustrate the feasibility, technical considerations, and clinical effectiveness of endoscope-assisted transmastoid infralabyrinthine approach (e-TIA).

## 2 Materials and methods

### 2.1 Anatomic study

**Fig. 1:**
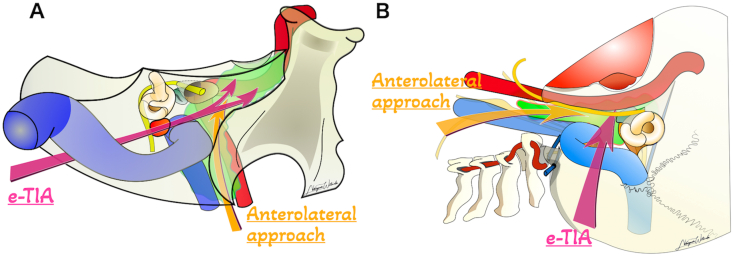
**Schematic illustrations demonstrating the concept of the endoscope-assisted transmastoid infralabyrinthine approach (e-TIA) in conjunction with the anterolateral approach.** Pink arrows indicate the surgical corridor of e-TIA, while orange arrows represent that of the anterolateral approach. The green area indicates a jugular foramen tumor with extension to petrous apex and cervical region. (A) Posterior view and (B) lateral view.

To establish the feasibility and technical details of e-TIA, we conducted an anatomical study. A schematic overview of this approach is illustrated in Fig. 1. The anatomical study was approved by the ethical committee of the Lariboisière Hospital. Dissections were performed in the experimental neurosurgery laboratory, Department of Neurosurgery, Lariboisière Hospital. Five cadaveric specimens (four left-sided and one right-sided) with latex-injected arteries and veins were used. High-resolution CT scans (slice thickness, 0.6 mm) were obtained at four stages: prior to dissection, following placement of the green molding clay at the petrous apex, following mastoidectomy (pre e-TIA), and after the completion of the procedure (post e-TIA). The CT images were imported into a neuronavigation system (StealthStation S7; Medtronic, Minneapolis, MN, USA) to identify the anatomical structures. Surgical microscope (Leica Camera AG, Wetzlar, Germany) and standard microsurgical instruments were utilized to perform the initial phases of dissection. For the endoscopic part of the dissection, a 30° endoscope with a 4-mm diameter lens (Karl Storz GmbH & Co. KG, Tuttlingen, Germany) was then employed in combination with a rotatable malleable suction instrument (Bissinger Medizintechnik GmbH, Teningen, Germany) Bone drilling was performed with bended diamond drill with self-irrigating protecting sleeve (Midas Rex MR8 high speed drill system, Clearview; Medtronic, Minneapolis, MN, USA).

#### 2.1.1 Placement of landmark at the petrous apex through transnasal route

**Fig. 2:**
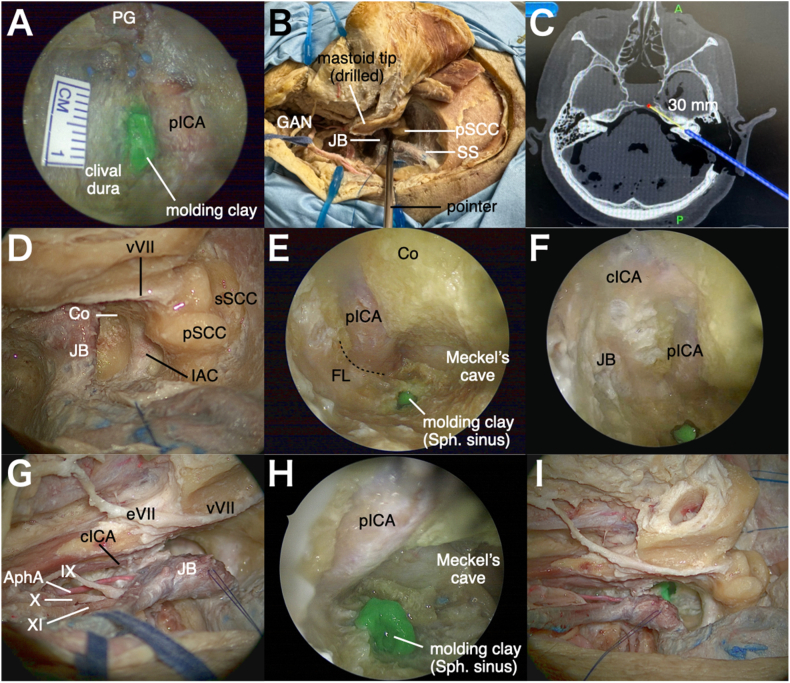
**Cadaveric dissection illustrating a left-sided e-TIA.** A: Transnasal view. A 5 mm green molding clay was put at the petrous apex on the left side. B: View after preliminary exposure using the anterolateral approach and partial mastoidectomy to identify the vertical segment of the facial nerve, the posterior semicircular canal, and the jugular bulb. C: A screen shot on Navigation showing direction of the probe with extension of 30 mm. D: Extensive bone drilling inferior to the posterior semicircular canal along the dura mater of the internal auditory canal until the cochlea. E: Opening of jugular fossa and posterior transposition of jugular bulb. F: A view toward rostrally with 30° endoscope after drilling of inferior part of the petrous bone, demonstrating an access to Meckel's cave and petrous segment of ICA, as well sphenoid sinus (green molding cray). G: A view toward caudally with 30° endoscope showing cervical segment of ICA and membrane between ICA and jugular bulb. H: Insertion of 30° endoscope between cervical segment of ICA and IJV, offering an alternative line of sight to the Meckel's cave, petrous segment of ICA, and sphenoid sinus. I: Final view showing the two corridors: the e-TIA corridor and the cervical corridor. *Abbreviations*: PG, pituitary grand; pICA, paraclival internal carotid artery; GAN, greater auricular nerve; JB, jugular bulb; pSCC, posterior semicircular canal; SS, sigmoid sinus; vVII, vertical segment of facial nerve; Co, cochlea; sSCC, superior semicircular canal; IAC, internal auditory canal; cICA, cervical segment of internal carotid artery; eVII, extratemporal segment of facial nerve; IX, glossopharyngeal nerve; AphA, ascending pharyngeal artery; X, vagus nerve; XI, accessory nerve; pICA, petrous segment of internal carotid artery; FL, foramen lacerum; Sph. sinus, sphenoid sinus.

To delineate the surgical target and provide a visual reference for e-TIA, a 7-mm green molding clay landmark was placed at the superior aspect of the petrous apex using a transnasal, transsphenoidal route. A 30° endoscope was introduced through the right nostril to access the sphenoid sinus. The paraclival internal carotid artery (ICA), pterygosphenoidal fissure, and clival dura were carefully exposed. Using a navigation system, the petrous apex was precisely identified. The green molding clay was then positioned (Fig. 2A).

#### 2.1.2 Preliminary exposure

The specimens were placed in supine position with the head fixed in a Mayfield head holder. The head was rotated 60° with a slight extension. Neuronavigation was prepared. A skin incision crossing the mastoid process and then following the anterior border of the sternocleidomastoid muscle (SCM) was performed with preservation of the greater auricular nerve. An anterolateral approach was performed as previously described (George and Lot, 1995; Giammattei et al., 2020). The SCM, splenius, and longissimus capitis were detached from the mastoid process and occipital bone. The muscles were retracted postero-inferiorly and dissection proceeds along the medial border of the SCM. Transverse process of C1 was palpated. The cranial nerve (CN) XI was identified within the fat tissue beneath the SCM. The internal jugular vein (IJV) was identified and isolated in front of the SCM. The CN X, CN XII, ICA, and external carotid artery were isolated. The superior and inferior oblique muscles and levator scapulae muscle were detached from the C1 transverse process. The C1 transverse process was removed and the V3 and V4 segments of the vertebral artery (VA) were exposed. A partial mastoidectomy was performed to expose the vertical segment of facial nerve and posterior semicircular canal (Fig. 2B). Image guidance of navigation system was used to define the trajectory of the e-TIA, from the infralabyrinthine suprajugular space to the petrous apex (Fig. 2C).

#### 2.1.3 Cross-sectional area measurements based on 3D reconstruction

To assess the adequacy of the working space provided by the e-TIA, three-dimensional (3D) image analysis was conducted. CT scans obtained pre and post e-TIA were exported in Digital Imaging and Communications in Medicine (DICOM) format and imported into Amira 6.3 software (Thermo Fisher Scientific, Waltham, Massachusetts, USA). The two CT datasets were merged. After the semi-automatical segmentation using threshold range of 200–1000 HU, manual correction was applied to refine the bone contours and fill the internal structures. A subtraction between pre- and post-e-TIA datasets allowed isolation of the drilled petrous bone, which was then segmented to reconstruct a 3D model of the e-TIA corridor. A central axis of the corridor was generated, and cross-sectional areas perpendicular to this central axis were obtained.

### 2.2 Clinical cases

Clinical data and surgical videos of three patients with multicompartmental jugular foramen tumor extending to petrous apex in which e-TIA were reviewed. Two patients were operated at Lariboisière hospital and one at University Hospital of Zurich by the senior author (SF). Informed consent was obtained from all patients included in this study.

## 3 Results

### 3.1 Surgical description of e-TIA to the petrous apex

Following the preliminary exposure, the endolymphatic sac was peeled from the dura (Alcântara et al., 2025). Extensive bone drilling was carried out inferior to the posterior semicircular canal along the dura mater of the internal auditory canal until the inferior aspect of the cochlea was identified (Fig. 2D). The jugular fossa was opened, and the jugular bulb was transposed postero-inferiorly (Fig. 2E). Through the infralabyrinthine corridor, a 30° angled endoscope, high-speed bent drill, and rotatable malleable suction were inserted using the chopstick technique (Labidi et al., 2018). By drilling the inferior part of the petrous bone, caudal and ventral to the cochlea, the petrous segment of the ICA was first identified. As bone removal proceeded around the ICA, the Meckel's cave was exposed rostrally (Fig. 2F). Distal dissection along the ICA allowed exposure of foramen lacerum. Drilling of superior portion of petrous apex provided access to the sphenoid sinus, where a green molding clay had been pre-placed via the transnasal route (Fig. 2F). Directing the endoscope caudally reveals the cervical segment of the ICA and the membrane separating the ICA from the jugular bulb (Fig. 2G). A 30° angled endoscope was inserted between the cervical ICA and IJV, via a cervical dissection, providing alternative line of sight to the Meckel's cave, petrous segment of ICA, and sphenoid sinus (Fig. 2H). Two corridors with different line of sight—the e-TIA corridor and the cervical corridor—were established (Fig. 2I).

### 3.2 Anatomical analysis with CT scan

**Fig. 3:**
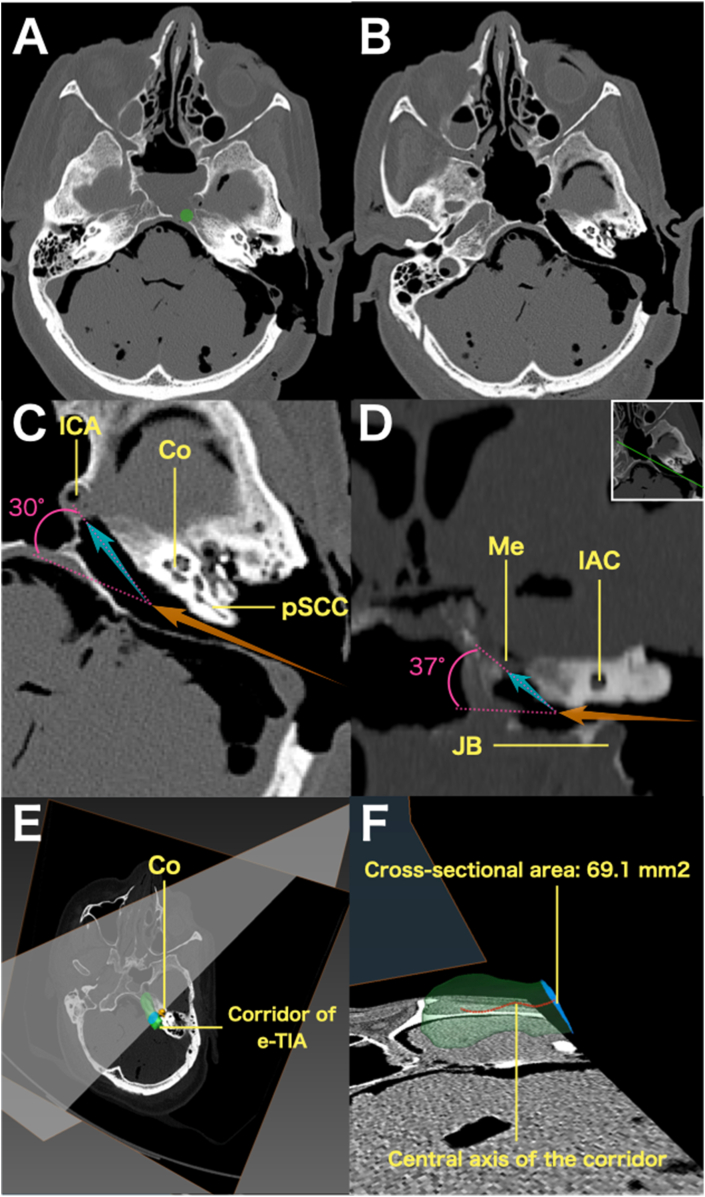
**The CT scan of the specimen 1.** A: Axial imaging following standard mastoidectomy and prior to e-TIA. Green circle indicates the location where a molding cray was placed transnasally. B: Axial imaging after e-TIA demonstrating access to the Meckel's cave, and to the petrous segment of ICA. C: Enlarged axial image post e-TIA. The orange arrow indicates the trajectory up to the cochlea, while the blue arrow shows the trajectory toward the lacerum segment of the ICA. The angular displacement between the two trajectories was 30°. D: Enlarged, tilted coronal image post e-TIA, corresponding to the cross-sectional plane shown in the upper right. The orange arrow indicates the trajectory toward the cochlea, and the blue arrow toward Meckel's cave. The angular difference between these trajectories was 37°. E, F: Three-dimensional reconstructed images created using Amira 6.3. The green object represents the segmented e-TIA corridor. A cross-sectional plane at the cochlear level, perpendicular to the central axis (red dashed line) is shown in blue. The cross-sectional area at this level measured 69.1 mm^2^. *Abbreviations*: ICA, internal carotid artery; Co, cochlea; pSCC, posterior semicircular canal; Me, Meckel's cave; IAC, internal auditory canal; JB, jugular bulb.

Fig. 3A and B demonstrate respectively the CT scans of pre and post e-TIA. Post e-TIA CT scan demonstrated the drilled corridor to the petrous apex passing dorsally to the cochlea. The location of the molding cray indicated by a green circle in Fig. 3A, was accessible through the e-TIA corridor (Fig. 3B). The Meckel's cave, the petrous and the cervical segment of ICA were successfully exposed (Fig. 3B). Beyond the cochlea, the trajectory changed by 30° in the axial plane, directing along the petrous segment of the ICA (Fig. 3C). In a coronal view, the trajectory toward Meckel's cave changed by 37° (Fig. 3D). This shift of trajectory justified the use of a 30° scope.

### 3.3 Cross-sectional area analysis

In all specimens, 3D reconstruction images based on CT scans demonstrated that the e-TIA corridor had an elongated configuration and the minimum cross-sectional area of this corridor was located at the level of the cochlea (Fig. 3E and F). The mean minimum cross-sectional area was 65.9 ± 12.6 mm^2^.

### 3.4 Illustrative cases

This endoscope-assisted approach was successfully applied in three patients. Below, we describe cases in detail to illustrate the utility and outcomes of this approach.

#### 3.4.1 Case 1

**Fig. 4:**
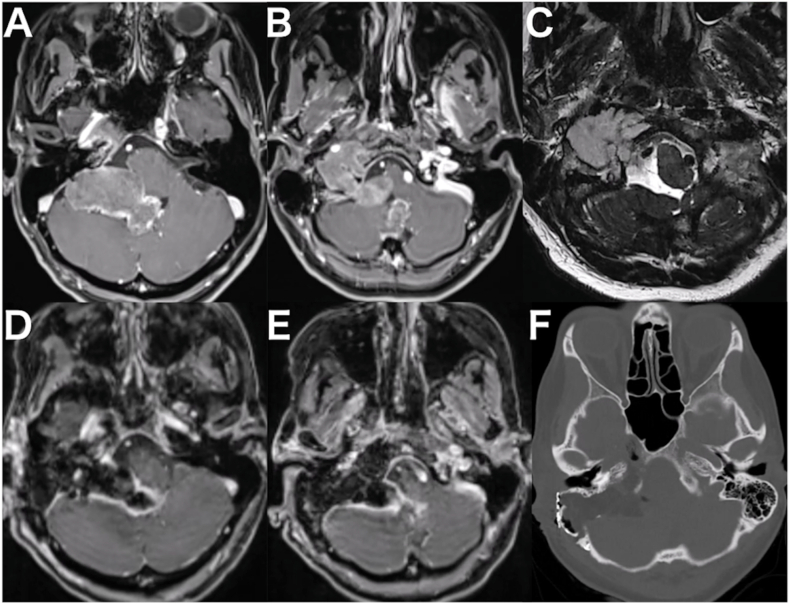
**The imaging studies of the case 1 in which right-sided e-TIA was performed.** A and B: Preoperative gadolinium enhanced T1-weighted MRI revealing the tumor with contrast enhancement extending from the petrous apex into the cervical and intracranial regions. C: Preoperative T2-weighted MRI demonstrating a hyperintense tumor. D and E: Postoperative Gadolinium enhanced T1-weighted MRI indicating near total resection. F: Postoperative CT scan showing adequate drilling of the petrous bone, including the petrous apex.

A 49-year-old female presented with a jugular foramen chondrosarcoma extending to the petrous apex and cervical region. A prominent intradural component caused brainstem compression, manifesting as progressive dizziness (Fig. 4A, B, and 4C). Surgical intervention was planned; however, the patient was urgently admitted due to escalating headaches, nausea, and vomiting. Intra tumoral hemorrhage resulted in fourth ventricle compression and obstructive hydrocephalus, necessitating an endoscopic third ventriculostomy. Following discharge to a rehabilitation facility, she was readmitted with worsening dizziness, recurrent vomiting, and severe swallowing difficulties. A follow-up CT scan indicated further displacement of the fourth ventricle, prompting a decision for tumor resection.

A combined right-sided e-TIA and anterolateral approach was performed incorporating intraoperative electrophysiological monitoring of multiple cranial nerves, including CN VII, IX, X, XI, and XII. A retroauricular skin incision crossing the mastoid process and following the anterior border of the SCM was made. Using anterolateral approach, IJV, ICA, CN X, XI, and XII, were exposed. The caudal part of the tumor, located in the cervical region below the jugular foramen, was partially resected. Following cosmetic mastoidectomy, the posterior semicircular canal, fallopian canal, and jugular bulb were exposed. When the tumor became visible in the infralabyrinthine space, the trajectory toward the petrous apex was confirmed using the navigation system (StealthStation S8, Medtronic, Jacksonville, FL, USA). Tumor resection began under 30° endoscopic visualization via the anterolateral corridor, allowing the removal of the extradural part into the jugular foramen. Subsequently, the approach was switched to e-TIA to address the intracranial tumor extending into the fourth ventricle and mixed with spontaneous hematoma. This component was carefully dissected from the cerebellum. Attention was then directed toward the part into the petrous apex: the cervical segment of the ICA was identified, and the pathological bone surrounding it was meticulously drilled following the ICA distally. This maneuver revealed the petrous segment of the ICA, Meckel's cave, and the sphenoid sinus. Navigation confirmed the full access to the petrous apex. Abdominal fat was put on the dural defect and within the mastoid cavity. The surgical procedure is demonstrated in Video 1.

Supplementary video related to this article can be found at https://doi.org/10.1016/j.bas.2026.105965

The following is/are the supplementary data related to this article:

Video 1 Case 1. Tumor removal using a combined right-sided e-TIA and anterolateral approach. Video 1

Histopathological analysis confirmed the diagnosis of grade II chondrosarcoma. Postoperatively, because of preoperative soft palate and right vocal cord paresis, a temporary tracheotomy was placed. Decannulation was achieved on postoperative day 7, and oral intake was resumed without significant aspiration. The patient experienced right-sided hypoacusis and tinnitus. She was subsequently transferred to a rehabilitation facility. Postoperative MRI showed near-total resection (Fig. 4D and E). Postoperative CT scan demonstrated adequate drilling of the petrous bone that facilitated tumor resection (Fig. 4F). At the two-month follow-up, the patient exhibited satisfactory oral intake. MRI demonstrated no evidence of tumor recurrence at two month and one year.

#### 3.4.2 Case 2

**Fig. 5:**
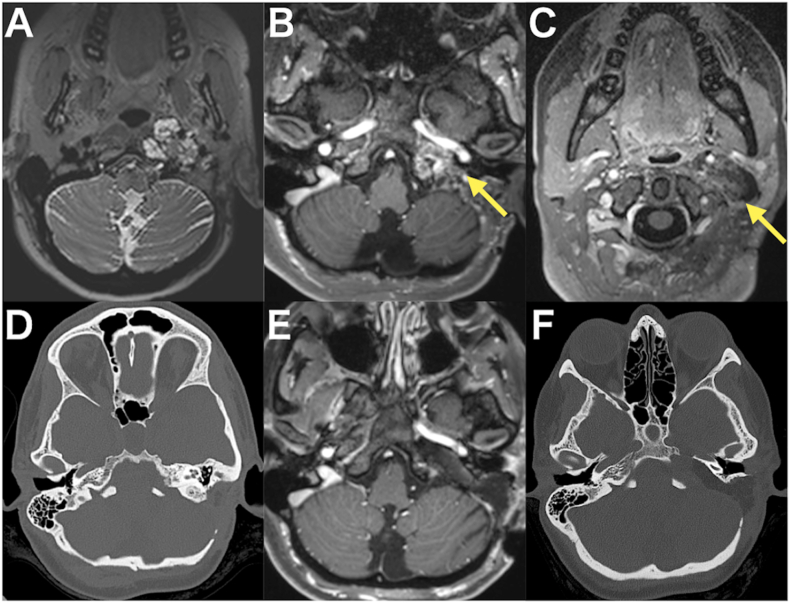
**The imaging studies of the case 2 in which left-sided e-TIA was performed.** A: Preoperative T2-weighted MRI showing the hyperintense jugular foramen tumor. B and C: Preoperative gadolinium enhanced T1-weighted MRI revealing the tumor with partial contrast enhancement extending from the petrous apex into the cervical region (arrows). D: Preoperative CT scan demonstrating pathological bone in the petrous bone. E: Postoperative gadolinium enhanced T1-weighted MRI confirming near-total tumor resection. F: Postoperative CT scan illustrating the surgical footprint, highlighting the drilled petrous apex.

A 29-year-old female presented with a left-sided jugular foramen meningioma. She had undergone two prior tumor resections. First surgery was a partial resection of the posterior tumor extension under the nuchal muscle. During her second surgery, an anterolateral approach with limited mastoidectomy was performed. Jugular foramen was opened with ligation of the sigmoid sinus and IJV to address intravenous tumor extension. Video of this second operation can be seen in a previous publication from the senior author (Giammattei et al., 2023). Histopathological examination revealed an unusual meningioma with papillary and rhabdoid features. A small residual tumor infiltrating the lower cranial nerves into the jugular foramen was left behind, which was treated with proton-beam therapy. Three years later, tumor regrowth was identified, and the patient presented with dysphagia and headaches. The tumor predominantly involved the petrous bone and extending anterior to the C1 and C2 (Fig. 5A, B, 5C, and 5D).

The resection was carried out with the electrophysiological monitoring of CN VII, IX, X, XI, and XII. Using an anterolateral approach, the IJV and ICA were exposed, revealing infiltration of the IJV. The tumor extending from the cervical region to the jugular foramen was microscopically resected. Subsequently, the residual mastoid bone from the previous surgery was drilled to expose the posterior semicircular canal, fallopian canal, and jugular bulb. Using the e-TIA, the internal auditory canal and cochlea were identified, and further drilling of the petrous bone under endoscopic visualization, allowed access to the tumor located at the petrous apex. After tumor resection and removal of the pathological bone, both the horizontal and vertical segments of the ICA, as well as Meckel's cave, were exposed. Introduction of a 30° endoscope through the anterolateral corridor allowed visualization of the trigeminal nerve within Meckel's cave. The surgical procedure is demonstrated in Video 2.

Supplementary video related to this article can be found at https://doi.org/10.1016/j.bas.2026.105965

The following is/are the supplementary data related to this article:

Video 2 Case 2. Tumor removal using a combined left-sided e-TIA and anterolateral approach. Video 2

Postoperative imaging showed near-total resection (Fig. 5E). CT scans confirmed extensive drilling of the petrous bone without compromising critical underlying bony structures (Fig. 5F). The postoperative course was uneventful, with no neurological complications. Preservation of hearing function was confirmed clinically. Dysphagia improved with physiotherapy, and the patient was discharged 11 days after surgery. Histopathological analysis revealed a meningioma with a minor papillary component and an inactivation of breast cancer 1-associated protein 1 (BAP1). She was treated with additional proton beam therapy with no sign of recurrence after one year.

#### 3.4.3 Case 3

A 41-year-old male with a history of chondrosarcoma had previously undergone subtotal resection via an anterior transpetrosal approach one year prior. Follow-up imaging revealed tumor regrowth, extending into the petrous apex, cervical region down to the anterolateral aspect of the C2 vertebral body, and along the lateral surface of the lower clivus.

Given the complex multicompartmental extension, a combined e-TIA and anterolateral approach was planned utilizing intraoperative monitoring of cranial nerves VII through XII. Surgical access to the cervical portion of the tumor was achieved first. The caudal tumor component was identified between the ICA and the IJV. A 30° endoscope was inserted along the tumor corridor, allowing resection to proceed rostrally until the jugular foramen and cervical segment of the ICA were exposed. Subsequently, the e-TIA was performed to address the petrous and clival components of the tumor. The petrous segment of the ICA was identified during drilling of the pathological bone. The resection proceeded in a caudomedial direction, enabling removal of the lesion invading the lower clivus. The inferior petrosal sinus and lower clivus were exposed, confirming the extent of access.

A gross total resection was achieved without postoperative complications, including hearing impairment or new cranial nerve deficits. The patient subsequently underwent adjuvant proton beam therapy. At two years postoperatively, he remains stable, with no radiological evidence of recurrence.

## 4 Discussion

This study evaluated the feasibility of extending the transmastoid approach to achieve comprehensive access to the petrous apex with preliminary clinical application in three illustrative cases. Our method provides an option in selected multicompartmental jugular foramen tumors (chondrosarcomas, meningiomas, glomus jugulare tumors) in which, traditionally, additional approaches or staged surgery would have been indicated to remove part of the lesion extending into the petrous apex.

The transmastoid infralabyrinthine approach was initially developed for drainage or biopsy of cystic lesions, such as cholesterol granulomas, within the petrous bone (Cömert et al., 2014; Schein et al., 2024; Wilson and Hodgson, 1991). Over time, adaptations of this corridor have facilitated tumor removal. Several critical factors in transmastoid approaches have been explored in prior studies. First, sufficient vertical width of the infra-labyrinthine supra-jugular space (distance between the posterior semicircular canal and the jugular bulb) is important for creating a surgical corridor to the petrous apex (Cinibulak et al., 2024a). Second, removal of bone around the jugular bulb and decompression increase the vertical width and broaden the angle of access to the petroclival region (Miller et al., 2016). Next, Anterior rerouting of the facial nerve has been employed to provide wider access from the retrofacial space to the jugular fossa (Cinibulak et al., 2024b). While effective, this technique carries a substantial risk of facial palsy, with a study reporting high complication rates (Llorente et al., 2014). Alternative approaches avoiding rerouting have been developed to minimize the risk of facial nerve injury. One technique involves removal of the mastoid tip anterior to the fallopian canal, combined with drilling of infratympanic and pretympanic bone, to access the cervical segment of the ICA. Another technique is referred as the “fallopian bridge” (Nonaka et al., 2013). Overall, reliance on a microscope alone requires extensive drilling to obtain an optimal line of sight and sufficient working corridor with a significant risk of facial palsy, reported to occur in up to 17.6 % of cases (Nonaka et al., 2013).

The use of an angled endoscope offers significant advantages over microscope. Unlike a microscope, which typically provides a direct line of sight, an angled endoscope offers additional perspectives for accessing deep-seated lesions. Shin et al. demonstrated the effectiveness of the endoscopic transmastoid posterior petrosal approach for tumors located in the internal auditory canal, jugular foramen, and hypoglossal canal, achieving good tumor removal rates with minimal complications (Shin et al., 2020).

In the present study, the use of 30° angled endoscope was critical in expanding the surgical exposure. Furthermore, the chopstick technique (Labidi et al., 2018) proved to be effective particularly in this narrow corridor to reach the petrous apex tumor extension. Without the use of an angled endoscope, the access would be restricted to a limited part of petrous apex due to the obstructions by the posterior semicircular canal, jugular bulb, internal auditory canal, and cochlea (Fig. 2D). In our study, the use of the endoscope allowed to benefit, beyond the level of the cochlea, of a wide bony space, which can be drilled until the petrous and cervical segments of the ICA and Meckel's cave are reached, even so, in clinical cases, the space is most often already created by tumor invasion. The angular displacements of the line of sight—30° in the axial plane toward the petrous segment of the ICA (Figs. 3C) and 37° in the coronal plane toward Meckel's cave (Fig. 3D)—demonstrate the effectiveness and rationality of 30° angled endoscope. This enhanced visibility, combined with the use of a bent drill and rotatable malleable suction, is critical to navigate into the petrous apex. The 3D reconstruction image of the e-TIA corridor (Fig. 3E and F) illustrates the narrow and elongated nature of the surgical corridor. Cross-sectional area analysis revealed that the narrowest point was at the level of the cochlea, with a mean cross-sectional area of 65.9 ± 12.6 mm^2^ across five cadaveric specimens. Given the use of a 4-mm endoscope, a 3-mm suction, and a 3-mm drill tip, the combined cross-sectional area occupied by these three instruments was approximately 32 mm^2^. This remains within the available space of the corridor, suggesting that surgical manipulation is theoretically feasible. However, reaching the target at the petrous apex requires precise positioning of the three instruments within this narrow and offset corridor, in order to avoid sword conflict. The use of chopstick technique is therefore mandatory. This concept is analogous to instrument handling in endonasal endoscopic approaches to lateral skull base regions using the chopstick technique (Labidi et al., 2018) or through other narrow surgical corridors (Fava et al., 2025).

When considering various approaches to the petrous bone, the unique shape of the petrous bone—an elongated triangular pyramidal structure—must be taken into account. A line of attack that follows the long axis of the petrous bone is crucial, particularly when working within narrow corridors. The line of attack in e-TIA is approximately aligned with the long axis of the petrous bone, from posterior to anterior (Fig. 1A and B). In comparison, the extended endoscopic transnasal approach (EEA), which targets the petrous apex and jugular foramen, also follows a line of attack that aligns with the long axis of the petrous bone. Two entry routes into the petrous apex have been described with EEA: one lateral to the ICA and one medial to it. The lateral route, bounded by the ICA medially, the maxillary nerve laterally, and the petrous ICA inferiorly, provides an access to the superior part of petrous apex and Meckel's cave lesion (Kassam et al., 2009). Approaches medial to paraclival ICA have been described (Taniguchi et al., 2016; Coutinho et al., 2024), can also be extended to regions adjacent to the inferior part of petrous apex, the petroclival region, and the jugular tubercle. These approaches often require transmaxillary transpterygoid approach, which may result in the sacrifice of some endonasal structures. Another approach for accessing the petrous bone is the anterior transpetrosal approach through the Kawase rhomboid which also provide a “looking-down” view to the intradural petroclival region (Tomio et al., 2022; Kawase et al., 1991). Even in cases with intradural component, such as Case 1, choice of a retrosigmoid approach is generally not a rational option. This is because the majority of petrous bone tumors—particularly chondrosarcomas—are primarily extradural, and dural opening via the retrosigmoid route may unnecessarily increase the risk of cerebrospinal fluid leakage. Additionally, considering the tumor's origin, it is likely that the lower cranial nerves are displaced dorsally, which further argues against this approach.

The anterolateral approach used in all three cases provided access to the cervical extension of jugular foramen tumor. Additionally, one of the major advantages of the anterolateral approach is to offer an upward line of sight for jugular foramen lesions compared to transpetrosal approach to the jugular foramen which requires additional exposure and manipulation of the facial nerve. This “looking-up” can be increased, as shown in the presented cases with the use of angled endoscope which allowed direct visualization of Meckel's cave compared to the transmastoid route (Fig. 2H). While the corridor between the IJV and ICA is typically narrow, tumors itself most often create the working corridor for the endoscope and instruments.

As summarized in Table 1, the choice of approach for petrous bone tumors depends on the tumor's origin and its vectors of extension. While previous studies have described transmastoid infralabyrinthine corridors, the novelty of our strategy lies in the systematic extension to the superior petrous apex and the quantified, dual-corridor surgical space. By combining the e-TIA with an anterolateral approach, we provide a continuous and expansive corridor that facilitates the management of complex multicompartmental tumors extending from the craniocervical junction to the superior aspect of the petrous apex. Another advantage of this combined strategy is the comprehensive surgical control of the ICA, covering from the cervical segments up to the lacerum segment. This elongated and angulated line of attack is particularly suited for soft and aspirable tumors, as the endoscope facilitates direct visualization within these narrow corridors.

**Table 1:** Strategic selection of surgical approaches for petrous bone lesions.

Approach	Core target in the petrous bone	Surgical Coverage		Accessible ICA	
eTIA with anterolateral (our study)	Jugular foramen Jugular fossa	Extradural	Petrous apex, Craniocervical junction	Cervical to petrous segment	
		Intradural	Cerebellomedullary cistern		
Endoscopic endonasal	Petrous apex	Extradural	Jugular tubercle	Petrous to Paraclival segment	
		Intradural	Prepontine		
Anterior transpetrosal	Petrous apex	Extradural	-	Petrous segment	
		Intradural	Prepontine/cerebellopontine area, Meckel's cave		
Transmastoid with fallopian bridge	Jugular fossa	Extradural	Facial recess	Cervical segment	
		Intradural	-		
Retrosigmoid	-	Extradural	-	-	
		Intradural	Cerebellopontine cistern Cerebellomedullary cistern		

However, anatomical constraints must be considered. A high jugular bulb remains a significant limitation, as it restricts the infralabyrinthine corridor. Furthermore, as landmarks become sparse beyond the cochlea, the integration of real-time neuronavigation is mandatory for the safe identification of the ICA, Meckel's cave, and the sphenoid sinus.

## 5 Conclusion

This study delineates the anatomical feasibility and technical nuances of the e-TIA based on cadaveric dissections and preliminary clinical applications. This endoscopic transmastoid corridor facilitates access to the superior aspects of the petrous apex, Meckel's cave, and the ICA from its cervical to lacerum segments. Despite its narrow, elongated, and angulated trajectory, cross-sectional area analysis confirms sufficient space for the simultaneous manipulation of an endoscope, suction, and drill using the chopstick technique. The integration of a 30° angled endoscope, curved drills, and rotatable malleable suction devices significantly enhances visualization and maneuverability. Furthermore, the dual-corridor strategy, combining the e-TIA with an anterolateral approach, provides a unique “looking-up” line of sight to the petrous bone. Overall, the e-TIA represents a promising option in selected multicompartmental jugular foramen tumors with petrous apex extension.

## Funding

This research did not receive any specific grant from funding agencies in the public, commercial, or not-for-profit sectors.

## Declaration of competing interest

The authors declare that they have no known competing financial interests or personal relationships that could have appeared to influence the work reported in this paper.

## References

[bib1] Alcântara T., Justo J., Jiang T. (2025). Optimizing extradural exposure in the posterior petrosal approach: the role of endolymphatic sac peeling. Oper. Neurosurg..

[bib2] Cinibulak Z., Krauss J.K., Nakamura M. (2013). Navigated minimally invasive presigmoidal suprabulbar infralabyrinthine approach to the jugular foramen without rerouting of the facial nerve. Neurosurgery.

[bib3] Cinibulak Z., Al-Afif S., Nakamura M., Krauss J.K. (2022). Surgical treatment of selected tumors via the navigated minimally invasive presigmoidal suprabulbar infralabyrinthine approach without rerouting of the facial nerve. Neurosurg. Rev..

[bib4] Cinibulak Z., Poggenborg J., Schliwa S. (2024). Assessing the feasibility of the transmastoid infralabyrinthine approach without decompression of the jugular bulb to the extradural part of the petrous apex and petroclival junction prior to surgery. Acta Neurochir..

[bib5] Cinibulak Z., Martinez Santos J.L., Poggenborg J. (2024). Comparative anatomic analysis of neuronavigated transmastoid-infralabyrinthine approaches for jugular fossa pathologies: short anterior rerouting versus nonrerouting and tailored nonrerouting techniques. Oper. Neurosurg..

[bib6] Cömert E., Cömert A., Çay N., Tunçel Ü., Tekdemir I. (2014). Surgical anatomy of the infralabyrinthine approach. Otolaryngol. Head Neck Surg..

[bib7] Coutinho Da Silva M.B., Hernández Hernández V., Gupta P. (2024). Anteromedial petrous (Gardner's) triangle: surgical anatomy and relevance for endoscopic endonasal approach to the petrous apex and petroclival region. Oper. Neurosurg..

[bib8] Fava A., Jiang T., Vu T.H., Abbritti R., Froelich S. (2025). Transorbital eyebrow lacrimal keyhole approach for resection of a meningioma of the lateral wall of the cavernous sinus. Neurosurg. Focus: Video.

[bib9] George B., Lot G. (1995).

[bib10] Giammattei L., di Russo P., Penet N., Froelich S. (2020). Endoscope-assisted anterolateral approach to a recurrent cervical spinal chordoma. Acta Neurochir..

[bib11] Giammattei L., Passeri T., di Russo P., Froelich S. (2023). Anterolateral (juxtacondylar) approach with limited mastoidectomy to resect a jugular foramen meningioma. Acta Neurochir..

[bib12] Kassam A.B., Prevedello D.M., Carrau R.L. (2009). The front door to Meckel's cave: an anteromedial corridor via expanded endoscopic endonasal approach-technical considerations and clinical series. Neurosurgery.

[bib13] Kawase T., Shiobara R., Toya S. (1991). Anterior transpetrosal-transtentorial approach for sphenopetroclival meningiomas: surgical method and results in 10 patients. Neurosurgery.

[bib14] Labidi M., Watanabe K., Hanakita S. (2018). The chopsticks technique for endoscopic endonasal surgery–improving surgical efficiency and reducing the surgical footprint. World Neurosurg..

[bib15] Llorente J.L., Obeso S., López F., Rial J.C., Coca A., Suárez C. (2014). Comparative results of infratemporal fossa approach with or without facial nerve rerouting in jugular fossa tumors. Eur. Arch. Otorhinolaryngol..

[bib16] Miller M., Pearl M.S., Wyse E., Olivi A., Francis H.W. (2016). Decompression of the jugular bulb for enhanced infralabyrinthine access to the petroclival region: a quantitative analysis. J Neurol Surg B Skull Base.

[bib17] Nonaka Y., Fukushima T., Watanabe K. (2013).

[bib18] Schein A., Guigou C., Madkouri R., Bozorg Grayeli A. (2024). Intraoperative imaging and navigation of the petrous apex by infralabyrinthine route. Eur. Ann. Otorhinolaryngol. Head Neck Dis..

[bib19] Shin M., Hasegawa H., Miyawaki S. (2020). Endoscopic transmastoid posterior petrosal approach for locally aggressive tumors in the petrous part of the temporal bone involving the internal auditory canal, jugular foramen, and hypoglossal canal. J. Neurosurg..

[bib20] Taniguchi M., Akutsu N., Mizukawa K., Kohta M., Kimura H., Kohmura E. (2016). Endoscopic endonasal translacerum approach to the inferior petrous apex. J. Neurosurg..

[bib21] Tomio R., Horiguchi T., Borghei-Razavi H., Tamura R., Yoshida K., Kawase T. (2022). Anterior transpetrosal approach: experiences in 274 cases over 33 years. Technical variations, operated patients, and approach-related complications. J. Neurosurg..

[bib22] Wilson D.F., Hodgson R.S. (1991).

